# Oregano Essential Oil Attenuates RAW264.7 Cells from Lipopolysaccharide-Induced Inflammatory Response through Regulating NADPH Oxidase Activation-Driven Oxidative Stress

**DOI:** 10.3390/molecules23081857

**Published:** 2018-07-26

**Authors:** Chuanshang Cheng, Yi Zou, Jian Peng

**Affiliations:** 1 Department of Animal Nutrition and Feed Science, College of Animal Science and Technology, Huazhong Agricultural University, Wuhan 430070, China; chengcs1989@gmail.com (C.C.); juicewaterzouyi@gmail.com (Y.Z.); 2 The Cooperative Innovation Center for Sustainable Pig Production, Wuhan 430070, China

**Keywords:** oregano essential oil, inflammation, oxidative stress, NADPH oxidase

## Abstract

Oregano is an aromatic plant widely distributed throughout the Mediterranean area and in Asia. Recent studies have revealed that the anti-inflammatory effect of essential oil in this plant. However, the mechanisms underlying the therapeutic potential have not been well elucidated. This study determined whether oregano essential oil (OEO) exerts an anti-inflammatory effect on lipopolysaccharide (LPS)-treated murine macrophage cells (RAW264.7 cells) in vitro and elucidated the possible underlying molecular mechanisms. The results showed that OEO (2.5–10 μg/mL) inhibited the expression and secretion of interleukin-1 beta (IL-1β), interleukin-6 (IL-6), and tumor necrosis factor-alpha (TNF-α) in RAW264.7 cells treated with LPS (1 μg/mL). Consistent with the pro-inflammatory gene expression, the OEO treatment efficiently reduced the LPS-induced activation of mitogen-activated protein kinase, protein kinase B, and nuclear factor κB in RAW264.7 cells. Nicotinamide adenine dinucleotide phosphate (NADPH) oxidase inhibition in Nox2 protein-silenced cells attenuated the mRNA expression of IL-1β, IL-6, and TNF-α in the LPS-induced RAW264.7 cells. The OEO inhibited the LPS-induced elevation of NADPH oxidase and oxidative stress. This result suggests that LPS induces RAW264.7 cell inflammation through the NADPH oxidase-mediated production of reactive oxygen species (ROS). In conclusion, OEO protects against the LPS-induced RAW264.7 cell inflammatory response through the NADPH oxidase/ROS pathway.

## 1. Introduction

Macrophages, which are ubiquitously distributed in tissues via the mononuclear phagocyte system, support homeostasis and host defense against intracellular parasitic bacteria and pathogenic protozoa [1]. Lipopolysaccharide (LPS), a component of the outer membrane of Gram-negative bacteria, stimulates macrophage activation [2]. Activated macrophages play an important role in inflammatory response by producing cytokines, including interleukin-1 beta (IL-1β), interleukin-6 (IL-6), and tumor necrosis factor-alpha (TNF-α) [1]. An inflammatory response is a crucial protective attempt of the host’s defense to remove injurious stimuli and initiate healing, but the overproduction of inflammatory mediators is a major cause of tissue damage, sepsis, and cancer [3].

Reactive oxygen species (ROS) overproduction and cell redox imbalance play a central role in the pathophysiology of the inflammatory response [4]. Substantial evidence implicates oxidative stress as an important pathogenic factor in inflammatory response [5]. ROS are intracellularly generated from several sources, including mitochondrial respiration, cytochrome P450, and the nicotinamide adenine dinucleotide phosphate (NADPH) oxidase system [6]. Phagocyte NADPH oxidase 2 (NOX2) is the major source of ROS generation in the macrophage response to LPS [7]. Therefore, LPS may induce an inflammatory response through NADPH oxidase activation-driven oxidative stress. Likewise, the inhibition of the ROS production using inhibitors against NADPH oxidase is proposed as an alternative approach to conventional antioxidant therapies against LPS-induced inflammation.

Oregano (*Origanum vulgare* L.) is an aromatic plant widely distributed throughout the Mediterranean area and Asia [8]. Oregano essential oil (OEO) is a concentrate of natural plant products that contain volatile aromatic compounds, and the major constituents of OEO have been characterized. This volatile aromatic compound exhibits several biological actions, including anti-bacterial and anti-oxidative activities [9]. Carvacrol and thymol, the two main phenolic derivatives that constitute about 81.9 and 3.5% of OEO, are principally responsible for the activities [9]. In addition, other minor constituents such as γ-terpinene and ρ-cymene, two monoterpene hydrocarbons that constitute about 4.49 and 3.07% of OEO, respectively, also contribute to the activities [10]. Recent studies have shown that OEO prevents inflammation in vitro and in vivo [11,12]. However, the regulatory mechanisms of OEO that reduce the inflammatory responses have not been reported. In the present study, we investigated the effect of OEO on the NADPH oxidase-mediated oxidative stress and the inflammation of murine macrophage cells (RAW264.7 cells) in response to LPS. We also determined whether the anti-inflammatory effect of OEO is associated with the inhibition of NADPH oxidase-derived ROS generation.

## 2. Materials and Methods

### 2.1. Chemicals and Reagents

Roswell Park Memorial Institute (RPMI) 1640 medium and fetal bovine serum (FBS) were purchased from GIBCO BRL (Grand Island, NY, USA). The oregano essential oil (OEO) was obtained from Meritech Bioengineering Co. Ltd. (Guangzhou, China), and its composition is shown in Supplementary Table S1 and Figure S1. The processing method of the OEO included mixing seeds, seedling cultivation, planting, harvesting, distillation and refining, quality inspection, qualified essential oil screening, and storage. The OEO was completely soluble in dimethyl sulphoxide (DMSO). The LPSs (*Escherichia coli* O127: B8) were from Sigma (St. Louis, MO, USA). The 2′,7′-dichlorofluorescein-diacetate (DCFH2-DA), diphenyleneiodonium chloride (DPI), and 3-(4,5-dimethyl-2-thiazolyl)-2,5-diphenyl-2*H*-tetrazolium bromide (MTT) were from Sigma-Aldrich (St. Louis, MO, USA). The anti-phospho p38 MAPK, anti-phospho anti-c-Jun N-terminal protein kinase (JNK), and anti-β-actin were from Cell Signaling Technology (Beverly, MA, USA). The anti-p38 MAPK, JNK, and anti-extracellular signal-regulated kinases 1/2 (ERK1/2) were from Affinity Biosciences (Columbus, OH, USA). The anti-proliferating cell nuclear antigen (PCNA) was from BD Transduction Laboratories (San Diego, CA, USA). The RAW264.7 cells were obtained from the Chinese Academy of Sciences Institution (Chinese Academy of Sciences, Guangzhou, China). The OEO was dissolved in the serum-containing medium to achieve the final desired concentration.

### 2.2. MTT Assay

The RAW264.7 cells (2 × 10^4^ per well in a 96-well plate) were washed with phosphate-buffered saline (PBS) and then added to 10 μL of 5 mg/mL MTT (dissolved in PBS). After being incubated at 37 °C for 4 h, the MTT solution was carefully removed and the resulting formazan crystals were dissolved in 150 μL of DMSO. Th absorbance was measured using a spectrophotometer (Biomate 5, Thermo Electron Corporation, Waltham, MA, USA) at the wavelength of 490 nm. The effect of OEO and LPS on the cell viability was assessed as the percentage of viable cells compared with the vehicle-treated control cells (received cell-grade DMSO at a safe concentration), which were arbitrarily assigned a viability of 100%.

### 2.3. Lactate Dehydrogenase (LDH) Release Assay

After the treatment period, the RAW264.7 cells culture medium was collected and assayed for LDH activity. The LDH release assay was detected using an LDH cytotoxicity assay kit (Nanjing Jiancheng Institute of Bioengineering, Nanjing, China), according to the manufacturer’s protocol.

### 2.4. Enzyme-Linked Immune Sorbent Assay for Quantification of Cytokines

The RAW264.7 cells (2 × 10^5^ cells per well in a 24-well plate) supernatants were centrifuged at 10,000× *g* at 4 °C for 10 min to remove debris, and stored at 80 °C until analysis. IL-1β, IL-6, and TNF-α were quantified using ELISA kits (R&D, Minneapolis, MN, USA), according to the manufacturer’s instructions.

### 2.5. Quantitative PCR

The total RNA from the RAW264.7 cells (2 × 10^5^ cells per well in a 24-well plate) was isolated using a Trizol reagent (Invitrogen, Carlsbad, CA, USA), according to the manufacturer’s instructions. The concentration and integrity of the RNA were measured at a 260/280 nm ratio. For the cDNA synthesis, 2.5 μg of RNA was reverse-transcribed using the Prime Script RT reagent kit (Takara, Tokyo, Japan), according to the manufacturer’s protocol. The PCR primers were designed using Primer 5.0 software, and the primer sequences have been shown in Table 1. The β-actin gene was used as invariant housekeeping gene internal control. Real-time PCR was performed, as described in our previous study [13]. Briefly, the reactions were as follows: 50 °C for 2 min, 95 °C for 30 s for one cycle; then 95 °C for 5 s, 59 °C for 15 s, and 72 °C for 45 s for 40 cycles. The relative gene expression was quantified by the comparative 2^−∆∆CT^ method [14]. All of the reactions were conducted in triplicate.

### 2.6. Western Blotting Analysis

The RAW264.7 cells (1 × 10^6^ cells per well in a 6-well plate) were collected and the total protein was extracted using a RIPA buffer containing protease inhibitors or phosphatase inhibitors (Thermo Scientific, Waltham, MA, USA). To determine the subcellular localization of nuclear factor kappa B (NF-κB) p65, nuclear extracts were prepared using a nuclear/cytosol fractionation kit (BestBio, Shanghai, China). A bicinchoninic acid (BCA) protein assay was used to determine the protein concentrations, and 30 μg of protein was loaded per sample. Western blotting was performed, as described in our previous study [13]. The chemiluminescence detection was performed using an ECL reagent (Thermo Scientific). Specific bands were detected, analyzed, and quantified by Image J Software (v 1.44, Bethesda, Rockville, MD, USA).

### 2.7. Measurement of ROS Generation

After the treatment period, the RAW264.7 cells (2 × 10^4^ per well in a 96-well plate) were washed with PBS and then incubated with 10 μM DCFH_2_-DA (dissolved in PBS). After incubated at 37 °C for 30 min, DCFH_2_-DA was removed and RAW264.7 cells were washed in PBS. Intracellular ROS, as indicated by DCF fluorescence, was observed with a fluorescence microscope (Nikon, Minato ku, Japan). The fluorescence intensity was quantified using a multidetection reader (Biotek, Winooski, VT, USA) at the excitation 480 nm and emission 520 nm. The RAW264.7 cells supernatant of the ROS level was measured by chemiluminescence assay using luminol, as described in our previous study [13]. The results were expressed as relative light units (RLU).

### 2.8. Assessment of Lipid Peroxidation, Intracellular GSH and GSH: GSSG Ration, and NADPH Oxidase Activity

IPEC-J2 cells (1 × 10^5^ per well in a 6-well plate) were washed twice with cold PBS and harvested by a cell scraper. The harvested cells were lysed by sonication on ice, and the supernatant of the centrifugate (1300× *g* at 4 °C for 15 min) was collected. Lipid peroxidation was assayed by the measurement of the malondialdehyde (MDA) level, as described previously [13]. The glutathione (GSH) levels and GSH: oxidized glutathione (GSSG) ration were determined using a commercial kit (Nanjing Jiancheng Institute of Bioengineering, Nanjing, China). The NADPH oxidase activity was quantified using ELISA kits (R&D), according to the manufacturer’s instructions.

### 2.9. SiRNA Transfection in RAW264.7 Cells

The NOX2-siRNA primer was as follows: 5′-GAGUUUGGAAGAGCAUAAUTT-3′ (sense) and 5′-AUUAUGCUCUUCCAAACUCTT-3′ (anti-sense). The siRNA targeting the mouse NOX mRNA was obtained from Genepharma (Shanghai, China). The RAW264.7 cells were grown in 6-well plates (2 × 10^4^ cells/well). After beng cultured for 24 h, the cells were then subjected to transient transfection with either a 100 nM NOX2-siRNA or a scrambled duplex, using the siRNA transfection reagent Lipofectamine 2000, according to the manufacturer’s instructions. After a 6 h incubation, the medium was replaced with a regular medium and the cells were cultured for another 24 h before treatment.

### 2.10. Statistical Analysis

The statistical analysis was performed using Prism software (Prism 5.0, San Diego, CA, USA). The data were analyzed by Student’s *t*-test, Student–Newman–Keuls’s multiple-comparison test, or two-way ANOVA using SAS v 8.2 (SAS Inst. Inc., Cary, NC, USA). The Student–Newman–Keuls’s multiple-comparison test was used for comparisons among the groups, and the LPS and NOX2 protein siRNA were used as factors in the two-way ANOVA. The values are presented as means ± standard error of the mean (SEM), and those at *p* < 0.05 were considered significant. 

## 3. Results

### 3.1. OEO and LPS Induces Cytotoxicity in RAW264.7 Cells

The cytotoxic effects of OEO and LPS on RAW264.7 cells were evaluated using MTT and LDH release assays. Low doses of OEO (1.25–20 μg/mL) did not cause any cytotoxicity, whereas doses of 80–160 μg/mL for 24 h significantly reduced the cell viability and increased the LDH release compared with the untreated control cells (Figure S2a,b). Based on these cell viability data, subsequent experiments were performed using OEO concentrations of ≤10 μg/mL. The results of the RAW264.7 cell exposure to LPS are shown in Figure S2c,d. The cells treated with LPS (100 μg/mL) showed significantly reduced viability and increased LDH release compared with the untreated control cells. To minimize the cytotoxic effect, treatment with 1 μg/mL LPS was selected for the subsequent experiments.

### 3.2. OEO Reduces LPS-Induced Expression of Inflammatory Mediators in RAW264.7 Cells

**Figure 1:**
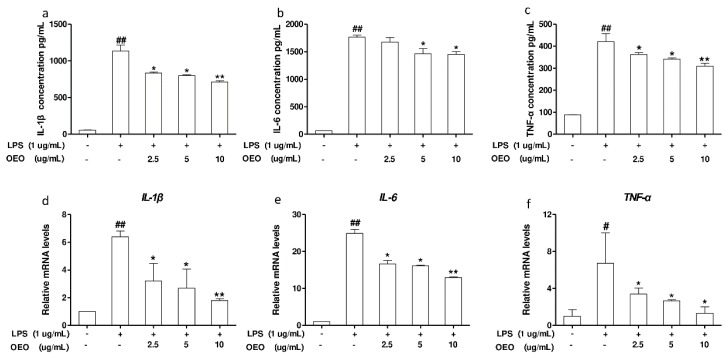
Oregano essential oil (OEO) inhibits lipopolysaccharide (LPS)-induced mRNA levels of pro-inflammatory cytokines expression in murine macrophage cells (RAW264.7 cells). (**a**–**c**) Cells were pretreated with OEO for 12 h, then incubated with 1 μg/mL LPS for 12 h. (**d**–**f**) Cells were pretreated with OEO for 12 h, then incubated with 1 μg/mL LPS for 1 h. The interleukin-1 beta (IL-1β), interleukin-6 (IL-6), and tumor necrosis factor-alpha (TNF-α) mRNA expression levels were measured using an RT-PCR analysis. Values represent means ± standard error of the mean (SEM), *n* = 3. ^#^ *p* < 0.05 and ^##^ *p* < 0.01 compared to the control group, while * *p* < 0.05 and ** *p* < 0.01 compared to the LPS-treated group.

As shown in Figure 1a–c, the production of IL-1β, IL-6, and TNF-α in RAW264.7 cells markedly increased in response to the LPS treatment for 12 h, whereas pretreatment with OEO (2.5–10 μg/mL) for 12 h significantly inhibited the secretion of IL-1β, IL-6, and TNF-α. Furthermore, we examined the mRNA levels of IL-1β, IL-6, and TNF-α through an RT-PCR analysis (Figure 1d–f). The treatment of RAW264.7 cells with LPS for 1 h dramatically induced the mRNA expression of IL-1β, IL-6, and TNF-α. Meanwhile, pretreatment with OEO (2.5–10 μg/mL) inhibited the mRNA expression of IL-1β, IL-6, and TNF-α in the RAW264.7 cells stimulated with LPS.

### 3.3. OEO Inhibits LPS-Induced Activation of Protein Kinase B (AKT), MAPKs, and NF-κB in RAW264.7 Cells

**Figure 2:**
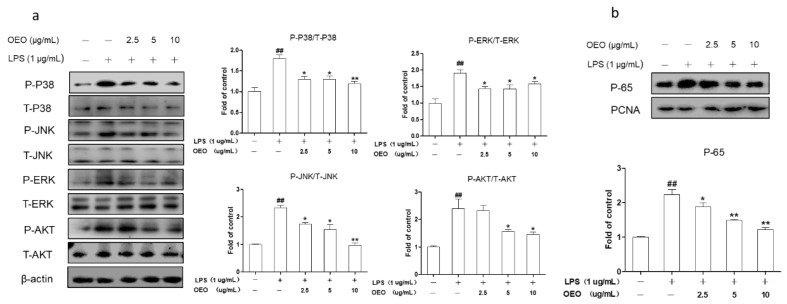
OEO inhibits LPS-induced AKT, MAPKs, and NF-κB activation in the RAW264.7 cells. (**a**) Cells were pretreated with OEO for 12 h, then incubated with 1 μg/mL LPS for 1 h. MAPKs and AKT were estimated by Western blot. (**b**) Cells were pretreated with OEO for 12 h, then incubated with 1 μg/mL LPS for 1 h. Western blot results show the effects of OEO on the protein levels of NF-κB p65 in the nuclear fractions. ERK1/2—extracellular signal-regulated kinases 1/2; JNK—c-Jun N-terminal protein kinase; P38—p38; Akt—protein kinase B; NF-κB—nuclear factor kappa B; P—phosphorylated; T—total. Values represent means ± SEM, *n* = 3. ^#^ *p* < 0.05 and ^##^ *p* < 0.01 compared to the control group, while * *p* < 0.05 and ** *p* < 0.01 compared to the LPS-treated group.

The production of inflammatory mediators is strongly affected by the AKT, MAPKs, and NF-κB pathways in the RAW264.7 cells. P65 is the major component of the NF-κB activated by LPS in the RAW264.7 cells. As shown in Figure 2, the RAW264.7 cells were pretreated with OEO for 12 h and stimulated with LPS for 1 h. The OEO suppressed the LPS-induced phosphorylation of AKT and MAPKs (Figure 2a), while decreasing the NF-κB p65 concentration in a dose-dependent manner in the nucleus (Figure 2b).

### 3.4. OEO Inhibits LPS-Induced Oxidative Stress in RAW264.7 Cells

**Figure 3:**
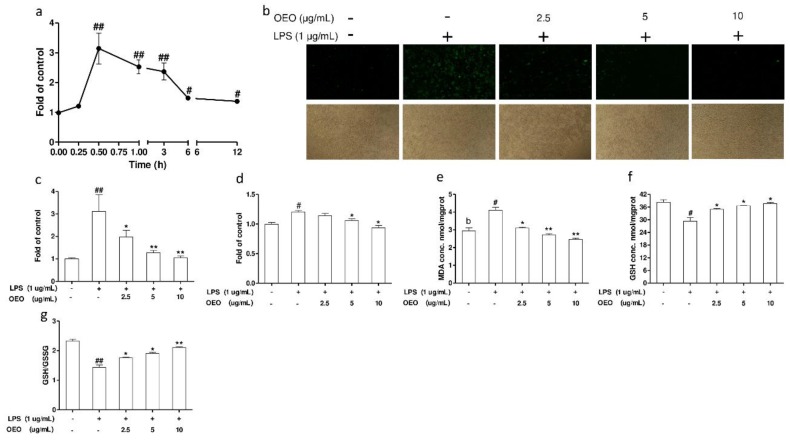
OEO inhibits LPS-induced oxidative stress in RAW264.7 cells. (**a**) The RAW264.7 cells were incubated with 1 μg/mL of LPS. The fluorescence intensity of the dichlorofluorescein (DCF)-stained cells was quantified using a flow cytometer. (**b**) Cells were pretreated with OEO for 12 h, then incubated with 1 μg/mL of LPS for 30 min. The intracellular reactive oxygen species (ROS) levels were indicated by DCF fluorescence and detected by fluorescence microscopy. (**c**) Cells were pretreated with OEO for 12 h, then incubated with 1 μg/mL LPS for 30 min. The fluorescence intensity of DCF-stained cells was quantified using a multidetection reader. (**d**) Cells were pretreated with OEO (2.5–10 μg/mL) for 12 h, then incubated with 1 μg/mL of LPS for 30 min. The levels of ROS in the culture supernatant were measured by chemiluminescence. (**e**–**g**) Cells were pretreated with OEO for 12 h, then incubated with 1 μg/mL of LPS for 12 h. The intracellular malondialdehyde (MDA), the glutathione (GSH) levels and GSH: oxidized glutathione (GSSG) ration were measured. Values represent means ± SEM, *n* = 3. ^#^ *p* < 0.05 and ^##^ *p* < 0.01 compared to the control group, while * *p* < 0.05 and ** *p* < 0.01 compared to the LPS-treated group.

The RAW264.7 cells were exposed to LPS for different time intervals (0.25–12 h). The increase in ROS generation started as early as 15 min, and the relative fluorescence peaked at 30 min, and subsequently maintained high levels at 6 and 12 h (Figure 3a). Intracellular ROS, as indicated by the DCF fluorescence, was observed with a fluorescence microscope. The RAW264.7 cells exposed to LPS for 30 min showed a significant increase in fluorescence (Figure 3b). This ROS induction was substantially inhibited by OEO pretreatment for 12 h (Figure 3c). The RAW264.7 cells’ supernatant of the ROS level was measured by luminol as a probe. The pretreatment with OEO for 12 h inhibited the ROS production in the RAW264.7 cell supernatants in the presence of LPS (Figure 3d).

The levels of malondialdehyde (MDA) were measured using the thiobarbituric acid reactive substances (TBARS) assay to investigate the effect of OEO on cell lipid peroxidation in cultured RAW264.7 cells. As shown in Figure 3e, the MDA levels in the RAW264.7 cells markedly increased in response to 24 h of LPS treatment, whereas pretreatment with OEO for 12 h significantly inhibited the lipid peroxidation. The exposure of RAW264.7 cells to LPS for 24 h decreased the GSH concentration and GSH: GSSG ratio in the cells. A pretreatment with OEO to the culture medium increased the GSH concentration and GSH: GSSG ratio in the cells (Figure 3f,g).

### 3.5. Inflammation Activation Induced by LPS Is Modulated by Nox2 in RAW264.7 Cells

**Figure 4:**
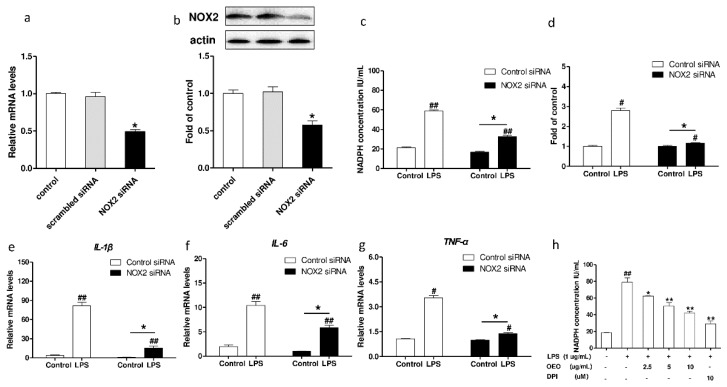
Effect of nicotinamide adenine dinucleotide phosphate (NADPH) oxidase 2 (NOX2) silencing on LPS-induced ROS production and inflammatory cytokines mRNA expression in RAW264.7 cells. (**a**,**b**) RAW264.7 cells were treated with NOX2-siRNA or control siRNA, and incubated for 24 h. (**a**) *NOX2* mRNA expression levels were measured using RT-PCR. (**b**) NOX2 protein expression levels were measured using Western blotting. (**c**) RAW264.7 cells were transfected with siRNA against NOX2 or control siRNA. After 24 h, the cells were incubated with LPS 1 μg/mL for 30 min. NADPH oxidases levels were measured using commercially ELISA kits. (**d**) RAW264.7 cells were transfected with siRNA against NOX2 or control siRNA. After 24 h, the cells were incubated with 1 μg/mL of LPS for 30 min. The fluorescence intensity of the DCF-stained cells was quantified using a multidetection reader. (**e**–**g**) RAW264.7 cells were transfected with siRNA against NOX2 or control siRNA. After 24 h, the cells were incubated with 1 μg/mL LPS for 1 h, and the IL-1β, IL-6, and TNF-α mRNA expression levels were measured using RT-PCR. (**h**) Cells were pretreated with OEO for 12 h or diphenyleneiodonium chloride (DPI) (10 μM) for 1 h, then incubated with 1 μg/mL LPS for 30 min. NADPH oxidases levels were measured using commercially available ELISA kits. Values represent means ± SEM, *n* = 3. ^#^ *p* < 0.05 and ^##^ *p* < 0.01 compared to the control group, while * *p* < 0.05 and ** *p* < 0.01 compared to the control-siRNA group (**a**–**g**) or the LPS-treated group (**h**).

NADPH oxidase is an enzymatic source for ROS production under various pathologic conditions. Nox2 is the catalytic subunit. DPI is a widely used inhibitor of NADPH oxidase [15]. The NOX2 protein was downregulated by treatment with NOX2-siRNA, as shown in Figure 4a,b. The treatment with NOX2-siRNA approximately reduced the mRNA and protein expression of NOX2 by 49% and 51%, respectively. The treatment with NOX2-siRNA suppressed the LPS-induced increase in the NADPH oxidase activity (Figure 4c) and the ROS production (Figure 4d). Moreover, the treatment with NOX2-siRNA inhibited the mRNA expression of IL-1β, IL-6, and TNF-α in the RAW264.7 cells induced by LPS (Figure 4e–g). The activity of the NADPH oxidase in the RAW264.7 cells increased in response to the LPS treatment for 30 min, whereas pretreatment with OEO or DPI significantly inhibited the activity of the NADPH oxidase (Figure 4h).

## 4. Discussion 

Macrophages, as innate immune cells, initiate inflammation and immune response [16]. LPS is the main component of endotoxin and is formed by a phosphoglycolipid or lipid A that is covalently linked to a hydrophilic heteropolysaccharide [17]. When challenged with LPS, macrophages are activated and release various pro-inflammatory factors, such as IL-1β, IL-6, and TNF-α [18]. The macrophage is activated as a defense mechanism, and is generally beneficial [19]. However, if the inflammation is uncontrolled, the excessive release may result in extensive tissue damage and pathological changes [20]. OEO exerts a significant anti-inflammatory effect in vitro and in vivo [12,21]. Our previous study found that OEO decreases the pro-inflammatory expression in pig models [21]. However, the molecular mechanisms by which OEO improves the anti-inflammation status are unknown. 

Importantly, the OEO concentrations (2.5–10 μg/mL) used in the present study were non-cytotoxic and extremely low. OEO at similarly low concentrations demonstrates favorable pharmacological properties. For instance, the treatment of oxidized low density lipoprotein-activated THP-1 macrophages with OEO (10–30 μg/mL) results in an overall reduction in pro-inflammatory cytokine release [12]. Similarly, the anti-inflammation effects of the OEO components thymol and carvacrol (3–6 μg/mL) have been demonstrated in high glucose-induced RAW264.7 cells [22]. To elucidate the mechanisms by which these anti-inflammation effects are exerted, we initially assessed the effects of OEO on the pro-inflammatory secretion in the RAW264.7 cells. In the present study, the most sensitive indicators of the effectiveness of the OEO treatment were shown to be the inhibited expression of the pro-inflammatory cytokines, such as TNF-α, IL-1β, and IL-6. Furthermore, LPS is a potent activator for various downstream signal transductions, including MAPK, Akt, and NF-κB [20,23]. The activation of these typical pathways is associated with an increased expression of TNF-α, IL-1β, and IL-6 [24]. Consistent with the inhibited expression of pro-inflammatory cytokines, the RAW264.7 cells treated with OEO showed a greater inhibition of the inflammation signaling pathway, including the MAPKs (e.g., p38 MAPK, ERK1/2, and JNK), Akt, and NF-κB signaling pathways.

ROS plays an important role in LPS-induced macrophage inflammation [25]. ROS induces inflammation by stimulating MAPKs, Akt, and NF-κB [26,27]. The model used in the present study increases the generation of free radicals and other ROS in the RAW264.7 cells [25]. The LPS treatment of the RAW264.7 cells dramatically increased the ROS production. Any imbalance between the production of these molecules and their safe disposal may culminate in oxidative stress. An in vitro study reported that OEO exerts an excellent anti-oxidative captivity by inhibiting lipid peroxidation and scavenging 2,2′-diphenyl-1-picrylhydrazyl radicals [28]. In the present study, OEO decreased ROS production in the RAW264.7 cells. MDA and GSH/GSSG are considered excellent indicators of oxidative stress [29,30]. OEO was beneficial in decreasing the MDA levels and increasing the GSH: GSSG ratio in the RAW264.7 cells induced by LPS. 

The phagocyte NOX2 complex is a multicomponent enzyme composed of membrane-bound subunits (Nox2 protein and p22phox) and cytosolic subunits (p47phox, p67phox, and Rac) [31]. The Nox2 protein acts as the catalytic subunit of the phagocyte NADPH oxidase [32]. NOX2 is highly expressed in phagocytes and in the RAW264.7 cells [31,33]. The NOX2 in the phagocytes constitutes the primary source of ROS during an oxidative burst [34]. The signal transduction and inflammatory response induced by LPS are related to the activity of NOX2 in a number of cells [35,36]. Previous results reported that LPS-induced superoxide formation and inflammatory are inhibited by silencing the Nox2 protein in human pulmonary microvascular endothelial cells [32]. In conjunction with our Nox2 protein silencing data, the NOX2-siRNA knockdown of the Nox2 protein inhibited not only the LPS-induced generation of ROS, but also its protective effect against oxidative stress-induced inflammation in the RAW264.7 cells. These results supported the role of Nox2-induced ROS production in LPS-mediated pro-inflammation expression. Therefore, the inhibition of ROS production using inhibitors against the NADPH oxidase is proposed as an alternative approach to conventional antioxidant therapies against inflammation. We examined the effect of OEO on the activation of NADPH oxidase in the RAW264.7 cells induced by LPS. The results showed that the OEO inhibited the ROS production in the RAW264.7 cells by reducing the NADPH oxidase activity. These data are consistent with previous observations that *Z. multiflora* essential oil, thymol, and carvacrol (1–100 ng/mL) significantly reduce the NADPH oxidase activity in the LPS-stimulated murine macrophage cells [37].

In summary, the results of this study showed that the LPS induced RAW264.7 cell inflammatory response through regulating NADPH oxidase activation-driven oxidative stress. OEO effectively inhibited the expression of the LPS-induced inflammatory mediators in the RAW264.7 cells through the activation of NF-κB and the phosphorylation of AKT and MAPKs via scavenging intracellular ROS. The OEO supplementation can effectively antagonize the NADPH oxidase activation, thereby abolishing the oxidative stress in the LPS-induced RAW264.7 cells. Our study indicates that dietary supplementation with OEO blocks LPS-induced RAW264.7 cell inflammation through decreasing the cellular levels of ROS, directly and indirectly.

## Figures and Tables

**Table 1:** Sequence information on the primers used for quantitative PCR. IL—interleukin; TNR—tumor necrosis factor.

Gene	Primer Sequence (Forward/Reverse 5′-3′)	Product Size (bp)
*IL-1β*	forward: ATGGCAACTGTCCCTGAACTCAACT reverse: CAGGACAGGTATAGATTCAACCCCTT	560
*IL-6*	forward: CCAGTTGCCTTCTTGGGACTGATG reverse: ATTTTCTGACCACAGTGAGGAATG	662
*TNF-α*	forward: ATGAGCACGGAAAGCATGATCCGA reverse: CCAAAGTAGACCTGCCCGGACTC	692
*β-actin*	forward: GGAGATTACTGCCCTGGCTCCTA reverse: GACTCATCGTACTCCTGCTTGCTG	101

## Supplementary Materials

Supplementary materials are available online, Figure S1: Typical chromatogram of oregano essential oil components, Figure S2: Oregano essential oil and LPS induced cytotoxicity in RAW264.7 cells, Table S1: Chemical composition of oregano essential oil components.
