# Supplementary material for: Oregano Essential Oil Attenuates RAW264.7 Cells from Lipopolysaccharide-Induced Inflammatory Response through Regulating NADPH Oxidase Activation-Driven Oxidative Stress
Source: Molecules. 2018 Jul 26;23(8):1857. doi: 10.3390/molecules23081857 (PMC6222776; doi:10.3390/molecules23081857)
Supplement: Supplementary file 1 [file molecules-23-01857-s001.pdf]

Supplementary Table S1. Chemical composition of oregano essential oil components.

ANALYSIS OF OREGANO OIL GC-MS

**Component**	**Oregano oil (MGZ-008) （%）**
α-Thujene/α-Pinene	0.56
Camphene	0.08
β-Pinene	0.09
Sabinene	0.03
Myrcene	0.91
α-Phellandrene	0.09
A-Terpinene	0.50
Limonene	0.15
1,8-Cincole+β-phellandrene	0.07
β-Ocimene	0.07
r-Terpinene	4.54
3-Ocimene	0.07
P-Cymene	3.11
Terpinoiene	0.05
3-Octanoi	0.11
1-Octen-3-ol	0.22
Dimethyl styrene	0.10
Trans-Sabinene hydrate	0.14
Linalool	0.32
cis-Sabinene hydrate	0.03
1-Terpincol	0.05
Terpinen-4-ol	0.22
Carvacrol methyl ether	0.33
B-Caryophyllene	1.43
Dihydrocarvone	0.09
α-Humulene	0.08
α-Terpineol	0.21
Borneol	0.33
β-Bisabolene	0.71
Caryophylene oxide	0.16
Thymol	1.90
Carvacrol	79.92

These results relate only to the sample(s) tested and do not guarantee the bulk of the mentioned to the equal quality.

Supplementary Figure S1. Typical chromatogram of oregano essential oil components.

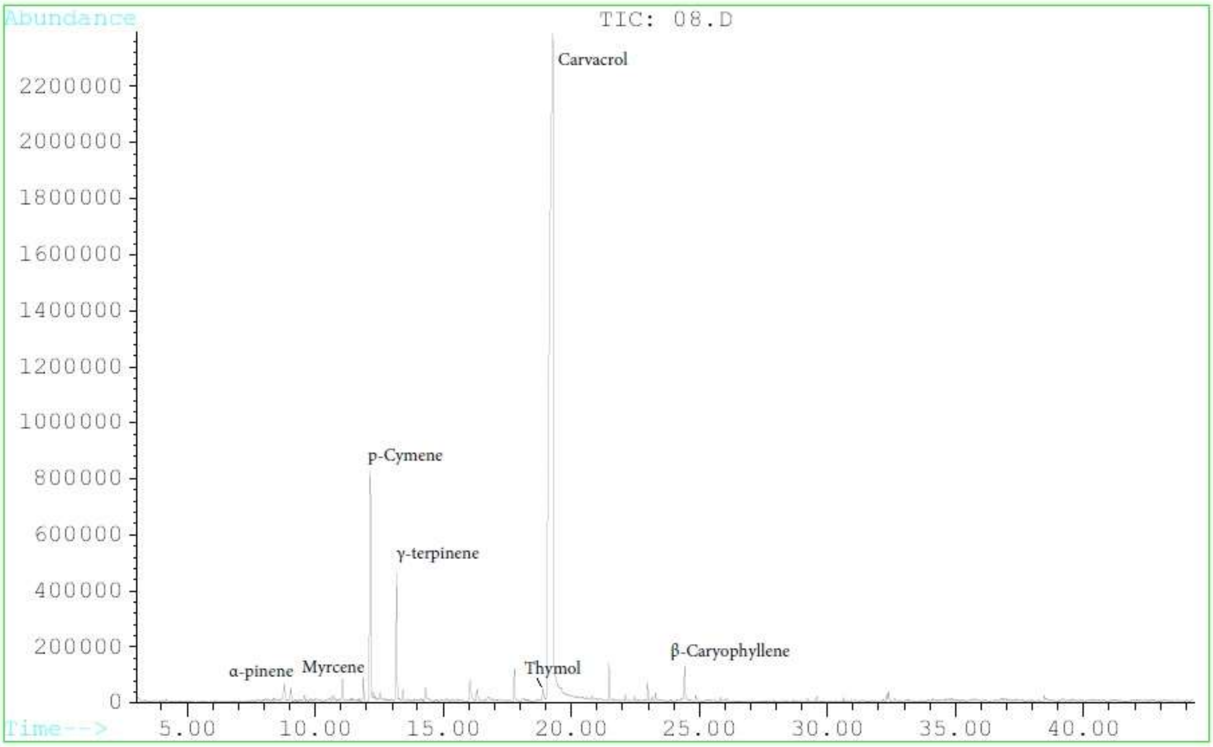

The Figure were provided by Meritech Bioengineering Co. Ltd.

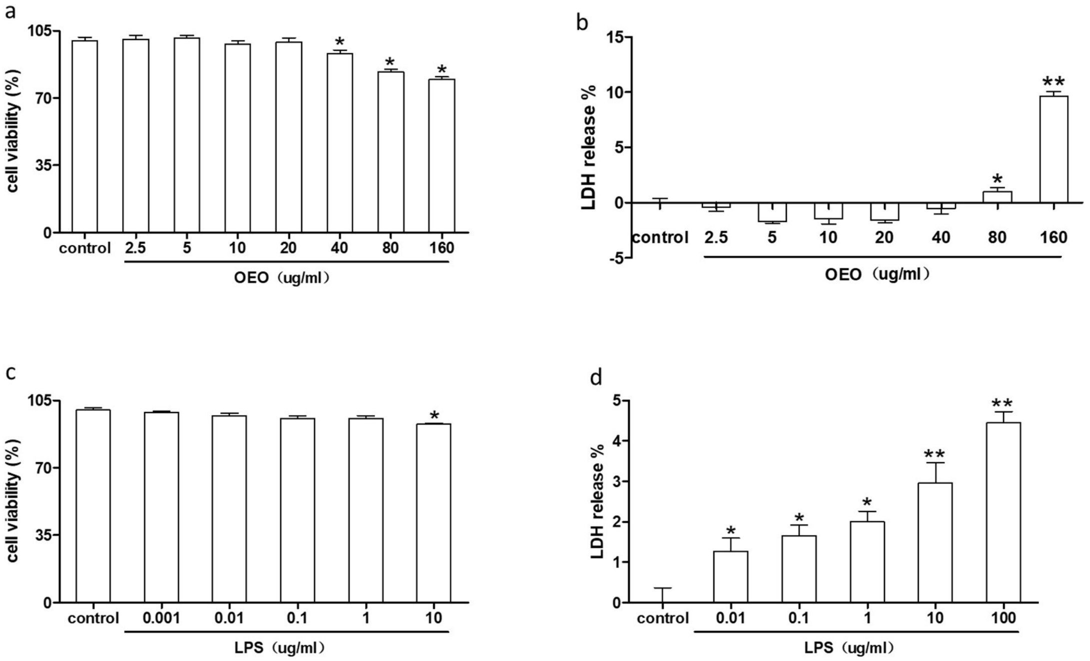

Figure S2. Oregano essential oil (OEO) and LPS induced cytotoxicity in RAW264.7 cells. (a) RAW264.7 cells were incubated with OEO (2.5–160 μg/ml) for 24 h. Cell viability was determined by MTT assay. (b) RAW264.7 cells were incubated with LPS (0.01-100 μg/ml) for 24 h. Cell viability was determined by MTT assay. (c) RAW264.7 cells were incubated with OEO (2.5-160 μg/ml) for 24 h. Cell cytotoxicity was determined by LDH release. (d) RAW264.7 cells were incubated with LPS (0.01-100 μg/ml) for 24 h. Cell cytotoxicity was determined by LDH release. Values represent means ± SEM, n = 3. *P < 0.05 and **P < 0.01 compared to the control group.
